# Supplementary material for: Combining Azimuthal and Polar Angle Resolved Shadow Mask Deposition and Nanosphere Lithography to Uncover Unique Nano-Crystals
Source: Nanomaterials (Basel). 2022 Oct 4;12(19):3464. doi: 10.3390/nano12193464 (PMC9565454; doi:10.3390/nano12193464)
Supplement: Supplementary file 1 [file nanomaterials-12-03464-s001.zip › nanomaterials-1918900-SI-done.pdf]

nanomaterials

Supplementary Information

Combining Azimuthal and Polar Angle Resolved Shadow Mask Deposition and Nanosphere Lithography to Uncover Unique Nano-Crystals

Arnab Ganguly and Gobind Das *

Department of Physics, Khalifa University, Abu Dhabi 12788, United Arab Emirates; arnab.ganguly@ku.ac.ae
* Correspondence: gobind.das@ku.ac.ae

S1. Matlab Simulation to Find Critical Value of φ

Shadow deposition of the nanosphere masks have been simulated using Matlab for different values of φ at constant value of θ (=0°). We observe that the area of masked region increases with increasing value of φ as shown in Fig. S1. At φ=45° the entire substrate is masked resulting in no metal deposition over the substrate at all. Thus, the critical value of φ is obtained as 45°.

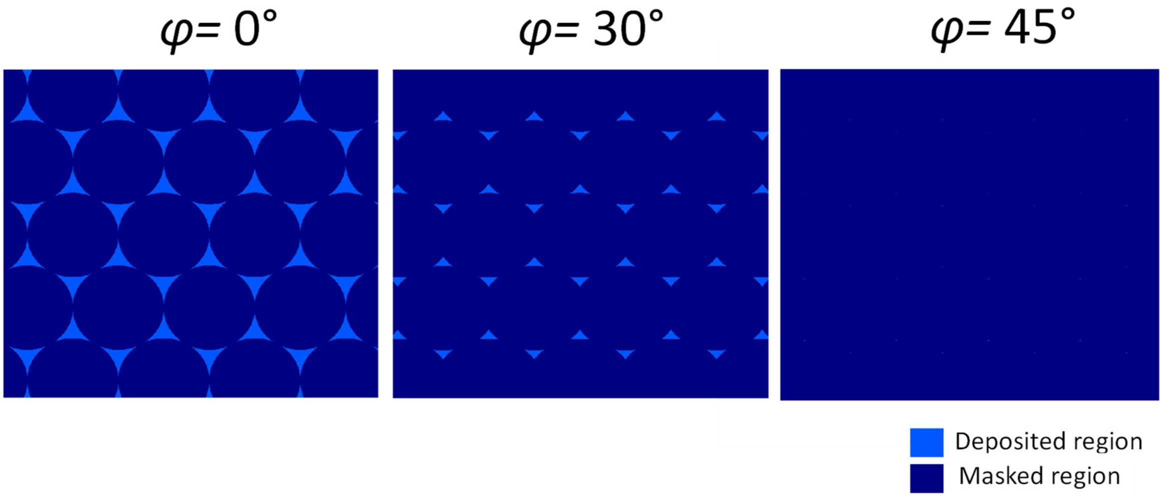

Figure S1. Simulation image of shadow deposition for θ =0° and φ=0°, 30° and 45°.

S2. Video of Continuous Variation of Tilt Angle without Azimuthal Rotation

Herein we attach an example video (vid_S2) of continuous variation of tilt angle φ at a constant azimuthal angle θ for a better understanding of the formation of 3D nanostructures discussed in Figure 2 in the main manuscript. In this video φ varies from 0° to 30° at constant θ = 20° giving rise to the final structure as shown in Figure 2a-Panel 3 in the main manuscript.

S3. Video of Continuous Azimuthal Rotation Angle with a Constant (or Varying) Tilt

Herein we attach example videos (vid_S3_a, vid_S3_b, vid_S3_c, vid_S3_d) of continuous azimuthal rotation with a constant (or varying) tilt for a better understanding of the formation of 3D nanostructures discussed in Figure 7 in the main manuscript.
